# Human Herpesvirus-6B Infection and Alterations of Gut Microbiome in Patients with Fibromyalgia: A Pilot Study

**DOI:** 10.3390/biom14101291

**Published:** 2024-10-12

**Authors:** Lauma Ievina, Nikita Fomins, Dita Gudra, Viktorija Kenina, Anda Vilmane, Sabine Gravelsina, Santa Rasa-Dzelzkaleja, Modra Murovska, Davids Fridmanis, Zaiga Nora-Krukle

**Affiliations:** 1 The Institute of Microbiology and Virology of the Riga Stradiņš University Science Hub, LV-1067 Riga, Latviamodra.murovska@rsu.lv (M.M.); 2 Latvian Biomedical Research and Study Center, LV-1067 Riga, Latvia; nikita.fomins@biomed.lu.lv (N.F.); dita.gudra@biomed.lu.lv (D.G.);; 3 Center for Neuroimmunology and Immune Deficiencies, LV-1050 Riga, Latvia; 4 Department of Biology and Microbiology, Rīga Stradinš University, LV-1007 Riga, Latvia; 5 Institute of Oncology and Molecular Genetics, Rīga Stradinš University, LV-1002 Riga, Latvia; 6 Department of Neurology, Pauls Stradiņš Clinical University Hospital, LV-1002 Riga, Latvia

**Keywords:** fibromyalgia, gut microbiome, HHV-6, biomarkers, cytokines

## Abstract

Fibromyalgia (FM) is a chronic disorder characterized by widespread musculoskeletal pain often accompanied by fatigue, sleep disturbances, memory issues, and mood disorders. The exact cause of FM remains unknown, and diagnosis is typically based on a history of persistent widespread pain, as there are no objective biomarkers usable in diagnosis of this disorder available. The aim of this study was to identify measurable indicators specific to FM with potential as biomarkers. This study included 17 individuals diagnosed with FM and 24 apparently healthy persons. Using real-time polymerase chain reaction (qPCR), we detected the presence of human herpesvirus (HHV)-6A and B genomic sequences in DNA isolated from peripheral blood mononuclear cells (PBMCs) and buccal swabs. HHV-6-specific IgG and IgM class antibodies, along with proinflammatory cytokine levels, were measured using enzyme-linked immunosorbent assay (ELISA) and bead-based multiplex assays. Additionally, the gut microbiome was analyzed through next-generation sequencing. HHV-6B was more frequently detected in the PBMCs of FM patients. FM patients with a body mass index (BMI) of 30 or higher exhibited elevated cytokine levels compared to the control group with the same BMI range. Gut microbiome analysis revealed significant differences in both α-diversity and β-diversity between the FM and control groups, indicating a shift in species abundance in the FM group.

## 1. Introduction

Fibromyalgia (FM) is a chronic condition characterized by widespread pain and tenderness in the muscles, ligaments, and tendons. It is often accompanied by other symptoms, including fatigue, sleep disturbances, cognitive difficulties, mood disorders, headaches, and irritable bowel syndrome. The estimated prevalence of FM is between 2% and 4% worldwide, with strong female predominance and higher percentages in risk groups [1]. FM heavily impacts the quality of life of patients and creates an economic burden on both individuals and the healthcare system [2,3]. The frequency of the disease increases with age, as well as symptom severity and worsening of quality of life [1]. 

Due to the lack of biomarkers for diagnostics, the primary method for diagnosing involves evaluating the severity of symptoms and considering the patient’s medical history [4,5,6]. Despite improvements in the criteria for assessing the disease, up to 75% of individuals with FM are still inaccurately diagnosed. This is due to the frequent overlap of symptoms with other conditions and the tendency for physicians to make subjective assessments rather than strictly following established guidelines [7]. Diagnostics are also challenged due to frequent additional health problems, as secondary FM is more common than primary FM. Patients with secondary FM frequently have infectious diseases and rheumatologic, neurologic, and mental disorders [8].

Results of multiple studies show that FM has a multifactorial origin [1]. Previous research shows a link between FM and viral infections [9,10]. Human herpesvirus (HHV)—6 and 7 infections are more prevalent in patients with FM than in apparently healthy individuals [11,12]. HHV6A and B both are members of the *Roseolovirus* genus, and the epidemiology of HHV6 in northern Europe can reach up to 97% [13,14]. Both of these viruses replicate in CD4+ T cells, and additionally, HHV6A can infect natural killer cells and γδT cells, while HHV-6B infects monocytes and dendritic cells [13]. Studies have shown an association between HHV-6 and several diseases involving the central nervous system (CNS), such as chronic fatigue syndrome/myalgic encephalitis, Guillain-Barré syndrome, and medial temporal lobe epilepsy [15,16]. Although the link between HHV-6 infection and the CNS is still unclear, the reactivation of the virus has been proven to induce proinflammatory cytokine production [17].

Altered cytokine levels could be considered as a potential biomarker for FM, given that chronic inflammation, pain conditions, and infections affect a wide range of cytokines [18]. However, results from previous studies on FM are often contradictory [19,20]. The most characteristic changes are associated with increased levels of pro-inflammatory cytokines such as interleukin-(IL)6, IL-8/CXCL8, tumor necrosis factor-alpha (TNF-α), interferon-gamma (IFN-γ), and decreased levels of anti-inflammatory cytokine IL-1 in the blood. FM patients also show elevated levels of C-reactive protein and brain-derived neurotrophic factor, which plays a crucial role in neuronal survival and growth [20].

Besides viral infections and changes in immune regulation, studies indicate that individuals with FM also experience altered gut microbial diversity and composition, as well as increased gut epithelial permeability [21,22,23]. These individuals show a decrease in beneficial bacterial genera such as *Lactobacillus*, *Bifidobacterium*, and *Eubacterium*, which are associated with a healthy microbiome [21]. The mentioned microbiome changes can affect both gastrointestinal symptoms, such as abdominal pain and irritable bowel syndrome, and impact the body outside the digestive tract. Microbiome-related metabolites penetrate into the bloodstream or release neurotransmitters that stimulate the vagus nerve, resulting in altered stress responses and behavior [24]. A recent systematic review of 92 observational studies aimed to explore the connection between rheumatic diseases and the gut microbiome. The review found differences in microbial diversity and dissimilarity metrics, along with a reduced number of short-chain fatty acid (SCFA) producing bacteria in FM patients [5]. This finding aligns with serum analyses showing significantly lower SCFA levels in FM patients compared to healthy controls [25].

Despite significant information in the literature on FM pathophysiology and potential diagnostic mechanisms, it remains insufficient, as there is no definitive diagnostic procedure based on biological biomarkers. Thus, our study aims to identify the prevalence of HHV-6 A and B infection and changes in pro-inflammatory cytokines and microbiome, as well as measure the potential link between these parameters. Our goal is to gain information about measurable indicators with a biomarker potential that could help diagnose and assess FM by analyzing viral infections, cytokine profiles, gut microbiome, and their interactions.

## 2. Materials and Methods

This study was approved by the Ethics Committee of Rīga Stradiņš University (permit No.: 2-PĒK-4/289/2022 and 2-PĒK-4/413/2024). Informed consent was obtained from all study participants prior to collection of samples and relevant clinical and phenotypical information. All activities within this study were conducted under biosafety level 2 and healthcare safety standards REGULATION (EU) 2017/745 OF THE EUROPEAN PARLIAMENT AND OF THE COUNCIL; 2017, https://eur-lex.europa.eu/legal-content/EN/TXT/?uri=CELEX%3A32017R0745 (accessed on 10 May 2024) [26].

### 2.1. Participants

As part of this pilot project, 17 patients (13 (76.5%) females, 4 (23.5%) males) with clinically diagnosed FM according to the 1990 American Rheumatology College FM diagnostic criteria were enrolled. Peripheral blood, feces, and buccal swab samples along with the information on symptom severity (by symptom severity score (SS), fatigue severity scale (FSS), and widespread pain index (WPI)), body mass index (BMI), age, and alcohol consumption habits were gathered from patients at the Center for Neuroimmunology and Immune Deficiencies. Genomic DNA from PBMCs, plasma, whole metagenome sequencing data, and category-wise matching phenotypical information of 19 apparently healthy age- and gender-matched persons were acquired from the “Genome Database of Latvian population” [27].

### 2.2. Genomic DNA Extraction

Blood samples were collected in vacutainers with K2EDTA and processed within 4 h of collection. Samples were centrifuged to isolate plasma and the Ficoll-Paque gradient method was used to separate PBMCs from other blood elements [28]. DNA from PBMC samples was extracted using an AllPrep DNA/RNA/Protein mini kit (Qiagen GmbH, Hilden, Germany) according to the manufacturers’ instructions, while DNA from buccal samples was isolated using the phenol-chloroform method. The concentration of DNA was measured employing a NanoDrop spectrophotometer (Thermo Fisher Scientific, Waltham, MA, USA), while the quality was assured by amplification of β-globin encoding gene fragment as described in Vandamme et al. [29].

### 2.3. HHV-6A and B Genomic Sequence Detection

RealStar HHV-6 PCR kit 1.0 (Altona Diagnostics, Hamburg, Germany) was used according to manufacturer instructions to detect HHV-6A and B genomic sequences in the DNA of PBMC and buccal swab samples.

### 2.4. Anti-HHV-6 IgG Detection

A semi-quantitative ELISA-VIDITEST anti-HHV-6 IgG (VIDIA, Prague, Czech Republic) kit was used according to manufacturer instructions to detect anti-HHV-6 IgG in plasma.

### 2.5. Cytokine Level Detection

The combination of MILLIPLEX^®^ Human Cytokine/Chemokine Magnetic Bead Panel—Immunology Multiplex Assay (Millipore, Sigma Aldrich, Darmstadt, Germany) and Luminex200 instrument following the manufacturers’ instructions was used to detect cytokine (IFN-γ, IL-1β, IL-2, IL-6, IL-8/CXCL8/CXCL8, IL-9, IL-17A/CTLA8, IL-18, TNF-α) levels in plasma samples.

### 2.6. Whole Metagenome Library Preparation and Next-Generation Sequencing

Whole metagenome library preparation was performed in accordance with a protocol previously published by Kalnina et al. [30]. All DNA from fecal samples was isolated using FastDNA SPIN Kit for Soil (MP Biomedicals, Irvine, CA, USA) according to the manufacturer’s recommendations. Quantification of DNA was carried out using a Qubit 2.0 fluorometer and Qubit dsDNA HS Assay Kit (both from Thermo Fisher Scientific, Waltham, MA, USA). Samples were then sheared using Covaris S220 Focused Ultrasonicator (Covaris, Woburn, MA, USA), and metagenomic library preparation was performed using MGIEasy Universal DNA Library Prep kit (MGI Tech Co., Shenzhen, China) according to the manufacturer’s instructions. Quality control of the libraries was performed using the Qubit dsDNA HS Assay Kit on a Qubit 2.0 instrument and using the Agilent High Sensitivity DNA Kit on an Agilent 2100 BioAnalyzer (Agilent Technologies, Santa Clara, CA, USA). FM group samples were sequenced using High-throughput Sequencing Set (FCL PE150), while control group samples were sequenced employing DNBSEQ-G400RS High-throughput Sequencing Set (FCL PE100) and DNBSEQ-G400 sequencer (all from MGI Tech Co., Shenzhen, China). Sequencing depth was calculated to achieve approx. 15 million paired-end reads per sample.

### 2.7. Sequence Analysis

To ensure comparability of sample sequences using two different sequencing lengths (100 and 150 bp paired-end), sample reads of the FM group were initially trimmed to 100 bp length using Trimmomatic v.0.39. The resulting raw sequencing reads were then subjected to quality filtering and adapter trimming using Atropos v.1.1.25 [31], where reads shorter than 75 nucleotides were excluded from further analysis. Reads were then compared to the human genome reference database (GRCh38/hg38) using the BMTagger v.3.101 program [32], and the complementary sequences were removed from the metagenomic data. Filtered reads were then taxonomically classified against the mpa_vOct22_CHOCOPhlAnSGB_202212 database using the Metaphlan4 v.4.1.0 tool [33] with the default parameters. Resulting per-sample data with absolute counts were merged into a single table using a custom Python v.3.12.4. script available at https://forum.biobakery.org/t/merge-metaphlan-tables-py-with-absolute-abundance/1839 (accessed on 19 March 2024). Lastly, using the R package mia v. 1.13.30 [34], the obtained microbial taxa abundance table was SRS-normalized at a depth of 15 million reads, and samples below this threshold were excluded from further analysis.

### 2.8. Statistical Analysis

Statistical analysis was carried out using the Jasp v.0.17.1.0. and R program version 4.3.2. Before the statistical analysis, the normal distribution of the data was assessed through descriptive statistics using the Shapiro–Wilk normality test. To determine the statistical significance of intergroup differences, the independent group non-parametric Mann–Whitney U test was employed. The non-parametric Spearman rank correlation coefficient was used to determine correlations. Results were considered significant if *p* ≤ 0.05.

The R package Mia was used to calculate Shannon and inverse Simpson indices that represent community diversity at the species level, and the statistical difference was evaluated using the Wilcoxon rank-sum test. Robust Aitchison rclr-transformed Euclidean distances [35] between samples were calculated using the phyloseq v.1.46.0 package [36] and visualized using principal component analysis (PCA). Homogeneity of variances between FM and the control group was evaluated using the PERMDISP test implemented in vegan v.2.6-4 package, whereas community-level multivariate comparisons were performed using permutational multivariate analysis of variance (PERMANOVA) using the adonis2 function from vegan. Differential abundance analysis of microbial taxa at the species level between groups was performed using the Zicoseq function implemented in GUniFrac v1.8 package [37], with a significance level of p_adj.FDR_ < 0.01. R packages ggplot2 v.3.5.0 and corrplot v.0.92 were used for the visualization purposes.

## 3. Results

### 3.1. Patients

The study group consisted of 17 patients with FM, of whom 13 (76.5%) were females and four (23.5%) were males. The mean age of the patients was 44 ± 10.9 years. The control group consisted of 24 practically healthy individuals, of whom 20 (83.3%) were females and five (20.8%) were males, mean age—43 ± 10.3 years. The average body mass index (BMI) in the FM group was 27.2 ± 5.4, while in the control group, it was 25.5 ± 5.

FM patients daily used 1–3 medications. Four (24%) of them used non-steroidal anti-inflammatory drugs such as ibuprofen, movalis, diclovit, and nimesil; four (24%)—antidepressants; five (29%)—anticonvulsants; and four (24%) patients used synthetic thyroid hormone L-thyroxine. Three (18%) patients used medications for high blood pressure. Among patients with FM, we noted the use of antipsychotic medications, synthetic glucocorticoids, opioid antagonists, as well as medications for treating polyneuropathy and type 2 diabetes.

### 3.2. Higher Prevalence of HHV-6 in Patients with FM Compared to Healthy Controls

**Figure 1:**
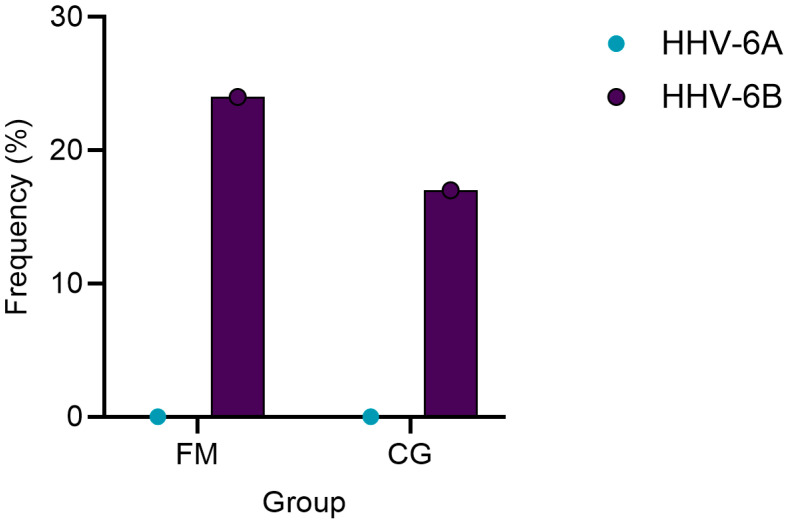
Frequency of HHV-6B detection in PBMC DNA among FM patients and control group. FM—patients with fibromyalgia; CG—control group.

Using real-time PCR, we examined the presence of the HHV-6 genomic sequence in DNA samples from PBMCs and buccal swabs. The results showed that HHV-6B was present in samples from seven (41%) FM patients (four (23.5%) in PBMCs and three (16.6%) in buccal swabs), while in the control group, it was found in the PBMC DNA of four (16.6%) individuals. Although the presence of the virus was more frequently detected in the case group than in the control group, the difference was not statistically significant (*p* = 0.6975) (Figure 1). Unlike HHV-6B, HHV-6A was not detected in any of the analyzed samples.

Quantitative results from qPCR showed that in all virus-positive samples, the absolute concentration of HHV-6B DNA was low, e.g., less than 10 copies per million PBMCs. On average, the concentration in the control group was 1.08, while in the FM group, it was even lower—0.38 copies per million PBMCs.

**Figure 2:**
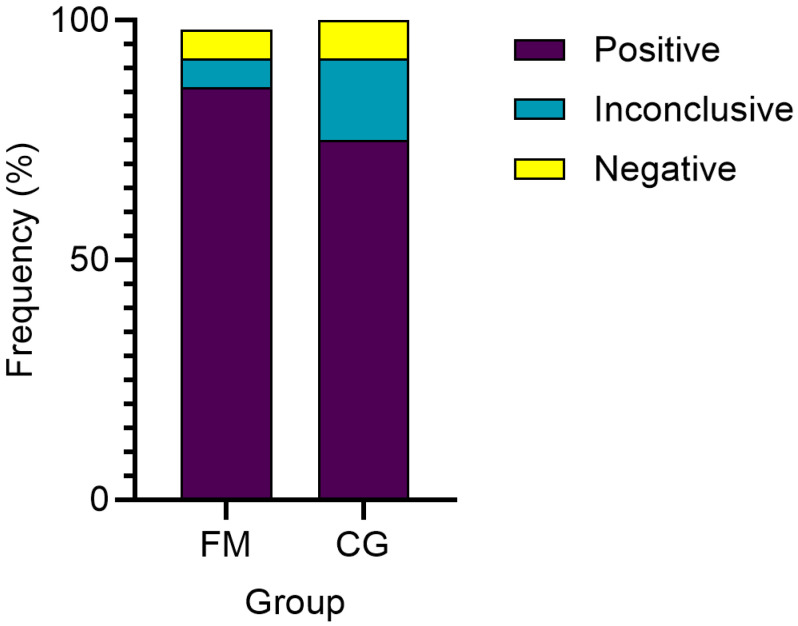
Comparison of anti-HHV-6 IgG detection frequency between FM patients and controls.

In contrast to the HHV-6B genomic sequence, the presence of anti-HHV-6 IgG in the plasma of participants from both studied groups was more frequently detected. Thus, these antibodies were detected in 88% of FM patients and in 75% of healthy individuals’ plasma samples. Only 6% of patients’ samples were negative, but 6% displayed inconclusive results. Similarly, in the control group, 8.3% of samples were anti-HHV6 IgG negative, and in 16.6% of samples, the result was inconclusive. A comparison of the groups showed that the differences in the number of positive samples were not statistically significant (*p* = 0.611) (Figure 2).

### 3.3. Similar Cytokine Levels in the Plasma of FM Patients and Healthy Controls

**Figure 3:**
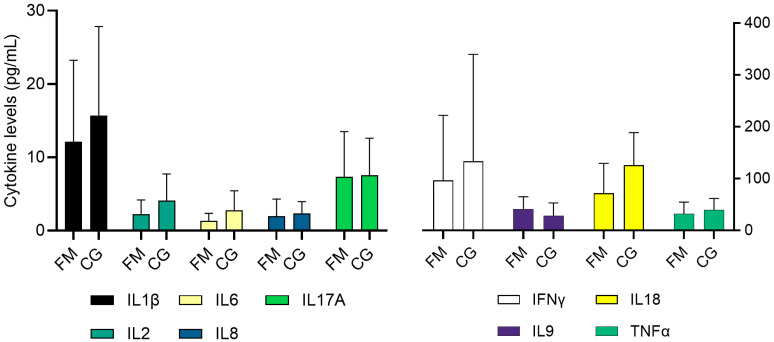
Comparison of cytokine plasma levels between FM and control groups (pg/mL).

When comparing cytokine levels in the plasma of FM patients and control individuals, we observed that most cytokines had higher levels in the control group. However, only the differences in IL-6 (*p* = 0.029) and IL-18 (*p* = 0.008) levels were statistically significant (Figure 3).

**Figure 4:**
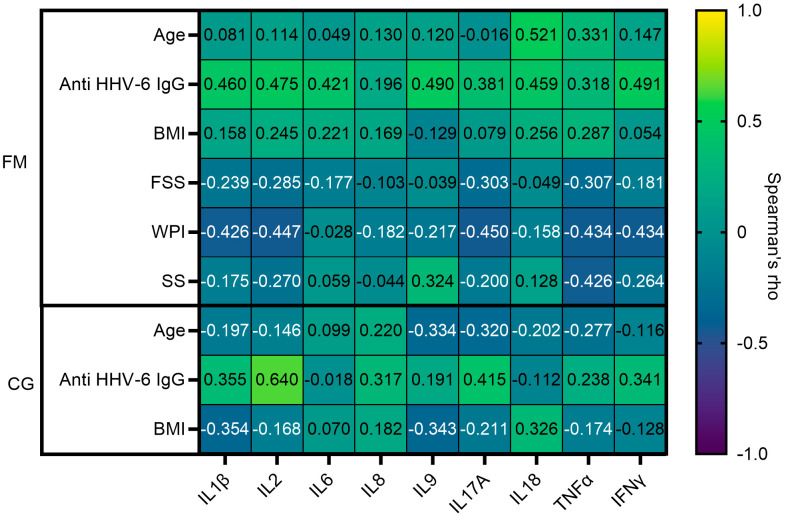
Correlation heatmap between cytokine levels and Anti HHV-6 IgG, Age, BMI, and FSS—Fatigue Severity score, WPI—widespread pain index, SS—symptom severity score.

Further, a comparison of cytokine levels between HHV-6B DNA-positive and negative FM patients revealed significantly elevated levels of TNF-α (*p* = 0.03) in those that were positive. In contrast, no HHV-6B infection-dependent association with cytokine levels was detected in the control group. To examine the relationship between variables such as age, gender, and BMI, we also performed a Spearman correlation analysis. Acquired results are summarized in Figure 4, which shows no statistically significant correlation between the tested variables and cytokines in either the FM or control group.

To better evaluate the impact of BMI on cytokine levels, we used this metric to divide participants of each group into two sub-groups. The first set of sub-groups was formed by participants with a BMI ≤ 29.9, while the second comprised participants with a BMI ≥ 30. Although statistically significant differences were not detected, we observed that there was a tendency for obese participants of the FM group (BMI ≥ 30) to have higher levels of IL-1β, IL-2, TNF-α, and IL-17A/CTLA8 (Table 1). Similarly, as in the patients’ group, we did not detect any statistically significant differences in the control group.

### 3.4. Gut Microbiome Composition

Sequencing analysis yielded on average 23,105,191.83 ± 9,062,264.44 (with median 23,736,333.5) high-quality sequences per sample, from which two samples of the FM group had atypically high levels of human sequences (65.97 and 74.26%, respectively) and relatively low numbers of detected microbial species (n = 94 and n = 86, respectively) and therefore were excluded from further analysis. Overall, gut microbiome samples were represented by 1566 bacterial species, and group CG was dominated by *Faecalibacterium prausnitzii* (4.36%), *Bacteroides uniformis* (4.24%), *Prevotella copri* clade A (4.03%) and *Fusicatenibacter saccharivorans* (2.44%), whereas the FM group was dominated by *Prevotella copri* clade A (12.35%), *Phocaeicola vulgatus* (5.54%), *Faecalibacterium prausnitzii* (4.34%) and *Bacteroides uniformis* (2.50%).

#### 3.4.1. Lower Microbial Diversity in Patients with FM than in Healthy Controls

**Figure 5:**
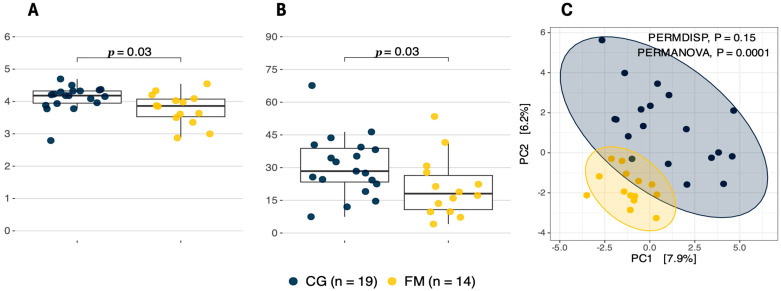
Community α- and ꞵ-diversity differences in gut microbiome at species level between CG and FM groups. Panel (**A**) represents the Shannon index measure; panel (**B**)—the inverse Simpson index, panel (**C**)—principal component analysis (PCA) with robust Aitchison distances.

A-diversity metrics revealed that both Shannon’s (*p* = 0.03), and inverse Simpson’s (*p* = 0.03) indices were significantly higher in CG individuals (4.11 ± 0.4 and 30.74 ± 13.9, respectively) compared to FM patients (3.78 ± 0.5 and 20.98 ± 13.7, respectively) (Figure 5). Further on, ꞵ-diversity analysis based on robust Aitchison distances revealed that there were no significant differences in dispersion homogeneity between CG and FM groups (*p* = 0.15, permutations = 9999); however, significant differences in ordinated centroids between these groups were identified (*p* = 0.0001, permutations = 9999), indicating global differences in bacterial taxa prevalence between the groups. Therefore, diversity analysis suggested that the taxonomical composition of the FM group was less diverse and more conserved compared to that of the CG individuals.

#### 3.4.2. Altered Bacterial Diversity in Patients with FM

**Figure 6:**
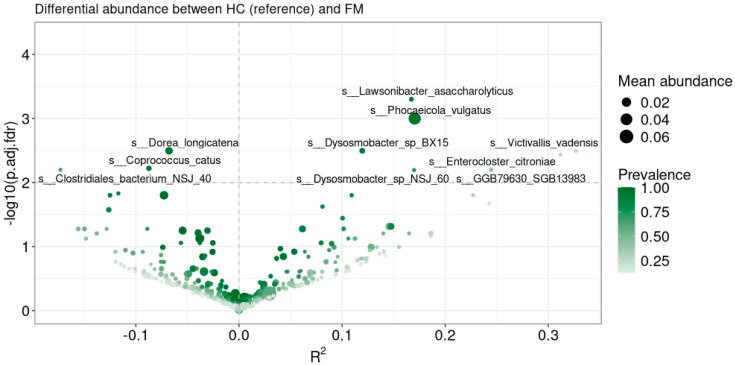
Volcano plot with differentially abundant taxa at species level between control group (CG) and fibromyalgia group (FM). The X-axis shows the effect size (negative: abundant in CG; positive: abundant in FM), and the y-axis shows the log_10_ false discovery rate (FDR)–adjusted *p*-values.

To uncover differences in bacterial abundance between FM and CG individuals, we conducted a differential abundance analysis. At a threshold of FDR-adjusted *p*-value < 0.01, we identified 10 species whose abundance differed significantly between the FM and CG groups, from which seven bacterial species were enriched and three depleted in gut microbiome samples of FM patients (Figure 6).

Depleted species in FM patient gut microbiomes included *Dorea longicatena* (FM = 0.32%, CG = 1.13%; p_adj_ = 0.0032), *Caprococcus catus* (FM = 0.055%, CG = 0.295%; p_adj_ = 0.0059) and *Clostridiales* bacterium NSJ 40 (FM = 0.0008%, CG = 0.014%; p_adj_—0.0064). Enriched species in FM patient gut microbiomes included *Lawsonibacter asaccharolyticus* (FM = 0.162%, CG = 0.029%; p_adj_ = 0.0005), *Phocaeicola vulgatus* (FM = 5.535%, CG = 1.299%; p_adj_ = 0.001), *Dysosmobacter* sp. BX15 (FM = 0.484%, CG = 0.103%; p_adj_ = 0.0032), *Victivallis vadensis* (FM = 0.094%, CG = 0; p_adj_ = 0.0032), *Enterocloster citroniae* (FM = 0.006%, CG = <0.0001%; p_adj_ = 0.0037), *Dysosmobacter* sp. NSJ 60 (FM = 0.042%, CG = 0.008%; p_adj_ = 0.0064), and GGB79630 SGB13983 assembled genome of *Firmicutes* phylum (FM = 0.037%, CG = 0.003%; p_adj_ = 0.0064).

## 4. Discussion

In this study, we compare the prevalence of HHV-6A and HHV-6B infection, cytokine levels in plasma, and changes in gut microbiome between patients with FM and healthy controls.

FM is a condition of unclear origin with various factors influencing its development. According to data in the literature, its manifestation is usually associated with bacterial and viral infections, severe and prolonged emotional stress, and genetic predisposition [1,38]. FM symptoms indicate that the disease primarily affects the autonomic nervous system [39]. The most common manifestations include widespread pain, joint stiffness, morning stiffness, sleep disturbances, and depression, as well as physical and mental fatigue [1]. Diagnosing FM is a complex and time-consuming process that often leads to an imprecise diagnosis [7]. It is based on the evaluation of the patient’s medical history and gathering of information through various questionnaires, such as the 1990 ACR diagnostic criteria, which rely on the presence of pain and symptom manifestations in affected individuals [6,40]. The results of previous studies on potential diagnostic biomarkers that are associated with FM were not conclusive, and thus far, a consensus in the field has not been reached [19,20].

One of the aspects that we evaluated in this study was the effect of HHV-6 infection on the onset of disease. However, low copy numbers of viral DNA in PBMC and buccal swab samples and the low concentration of anti-HHV-6B IgG indicated that in all positive participants, the viral infection was in a latent state.

According to our data, the frequency of anti-HHV-6 IgG-positive participants was 88% in FM patients and 75% in the control group, which is consistent with the data in the literature on the prevalence of anti-HHV-6 IgG in the population. Some of the previous studies on the local population have shown that 76.6% of healthy individuals in Latvia have HHV-6 virus-specific IgG antibodies in their blood plasma, which is in good agreement with the observed anti-HHV-6 IgG prevalence in the control group of this study [15]. In contrast to the general population, data in the literature on the prevalence of anti-HHV-6 IgG in FM patients are limited. However, existing publications contain no indications that there are differences in HHV-6 antibody prevalence between individuals with FM and healthy individuals, which is similar to our findings [41]. A study published in 2019 reported that the frequency of HHV-6 genome detection in both the FM group and the control group was 7% and 5%, respectively, which is in sharp contrast to our data, where the frequency was 23.5% vs. 16.6%, respectively [12]. However, our results indicating a statistically insignificant difference in HHV-6 prevalence between the FM and control groups are in line with those of international studies that also reported an association between HHV-6 infection and FM [11,12].

Since viral infections are often accompanied by changes in the regulation of immune response, we and several research groups also performed estimations of various cytokine levels. On the overall scale, the published results seem to indicate a tendency toward higher cytokine levels in FM patients [20]. Thus, 2021 and 2022 meta-analyses determined that FM patients typically have higher levels of IL-6, IL-8/CXCL8, TNF-α, IFN-γ and lower levels of IL-1. However, it should be noted that some of the meta-analyses’ studies displayed reversed observations in at least some of the variables [19,20]. Such changes in the mentioned inflammatory markers were not observed in this study; for example, IL-6 was significantly lower in the fibromyalgia group. These pronounced differences in results can be explained by the varying number of study participants, as the number of participants in the studies included in the meta-analyses varied by up to 255 participants [20]. Alternatively, the cause of such differences between our and other studies could be also induced by the differences in sampling strategy. In most of the studies that were included in the meta-analyses, participants were asked to go through the one-week medication ’washout’ period before sample donation, but such practice was not used in this study. As mentioned earlier, in the FM group, 24% of participants used non-steroidal anti-inflammatory drugs, and one participant used synthetic glucocorticoids—medications that reduce inflammation through inhibition of cytokine interactions, chemotaxis, and cyclooxygenase enzyme isoforms COX-1 and COX-2 as well as alteration of gene transcription processes [42]. Similarly, as in our study, lower levels of TNF-α and IL-9 in FM patients were observed during investigations that were conducted by Clos-Garcia et al. [21]. In this study, FM patients also continued to use their medications according to their needs and prescriptions before sample collection, thus confirming the influence of medication on cytokines [21].

However, when analyzing cytokine levels, it is essential to consider the effect of BMI, because increased body weight is often observed in individuals with FM. According to data in the literature, 60–70% of FM patients display increased body weight, which also fits well with our data [20]. People with obesity experience chronic inflammation due to the endocrine function of adipocytes, which leads to a release of such pro-inflammatory cytokines as IL-6, IL-1β, and TNF-α [43]. A similar increase in cytokine levels is also observed in cases of FM [20]. Although a significant correlation between BMI and cytokine levels was not observed in our study, the division of the participants revealed a tendency of increased IL-1β, IL-2, TNF-α, and IL-17A/CTLA8 levels in individuals with a BMI ≥ 30.

In their study, Ghafouri et al. observed a correlation between the cytokines IL-1RA and IL-6 and the results of the “Fibromyalgia Impact Questionnaire”, particularly in individuals with obesity. They found that a higher BMI and increased levels of these cytokines were associated with the exacerbation of symptoms in people with FM [44]. Similar associations between increased body weight and more pronounced FM symptoms, such as depression and pain, were also reported by other researchers [45]. However, similarly to our data, Okifuji et al. detected a positive correlation between BMI and IL-6 in individuals with FM, but without association between BMI and severity of symptom manifestation [46].

Exploring the gut microbiome composition of FM patients, we observed reduced diversity and an enrichment of Gram-negative bacterial species such as *Phocaeicola vulgatus*, *Victivallus vadensis* and *Enterocloster citroniae*, accompanied by a reduced abundance of Gram-positive species like *Dorea longicatena* and *Caprococcus catus*. The depleted species are known beneficial metabolite producers; for instance, *D. longicatena* produces indole, which helps to maintain the intestinal homeostasis by strengthening epithelial cells, suppressing inflammation, regulating gut insulin secretion, and promoting spore and biofilm formation [37,47]. Moreover, indole and its derivatives are associated with neurological and neuropsychiatric disorders and therefore are considered as key mediators in the gut–brain axis. Another depleted metabolite producer in FM patients is *C. catus*, which is a known propionate and butyrate producer [48]. These metabolites can reduce local and systemic inflammation, as well as maintain intestinal epithelial cell integrity [49]. In addition, in this study, two FM patients were excluded from the dataset due to a high level of human-origin sequences, possibly resulting from alterations in the levels of beneficial metabolite-producing bacteria, which might lead to increased intestinal permeability. In FM patient gut samples, an increased abundance was observed for *E. citroniae*—a bacterium associated with type 2 diabetes in the Finnish population [50] and found in higher levels in patients with chronic hepatitis B virus infection [51]. Additionally, an increased abundance *V. vadensis*, a common member of the gut microbiome with the ability to produce extracellular mucus, was observed in FM patients [52]. Although the detected discrepancies in species abundance between FM patients and generally healthy individuals are intriguing, a larger sample size is required to draw solid conclusions. Nevertheless, these results highlight the need for further research, incorporating metabolomics to investigate alterations in microbiome-produced metabolites.

Even though the results of DNA assays were corrected for sex differences, other potential confounders—beyond gender—should be evaluated in further studies, as they may affect the gut microbiome, such as medication and diet. Minerbi et al. evaluated the impact of supplementary medication on the gut microbiome but observed no significant effect [25]. Pregabalin and gabapentin, which are anticonvulsants commonly used in our FM cohort for symptom relief, may potentially affect gut microbiome composition and diversity. However, the effect of medication intake was not evaluated in relation to microbial metrics [53].

Future research could explore ways to minimize the impact of routine medication use by encouraging participants to temporarily discontinue medications prior to sample collection, where feasible. Additionally, incorporating data on daily habits, such as physical activity, may provide deeper insights into their effects on cytokine profiles and gut microbiome composition. Investigating the efficacy of disease-targeted physical activity programs by analyzing changes in clinical indices, physical measurements, proteomics, and biochemical markers could lead to the development of safer and more cost-effective treatments for FM. Furthermore, future studies might expand their focus to include analyses of additional human herpesviruses (HHVs), gut microbiome short-chain fatty acids, and control group symptom assessments to enhance the understanding of FM.

## 5. Conclusions

This pilot study indicates a tendency where HHV-6B genome sequences are more frequently found in FM patients accompanied by higher plasma levels of TNF-α, compared to healthy individuals.

Additionally, significant differences were observed in the Shannon α-diversity and β-diversity of the gut microbiome between FM patients and matched healthy controls, indicating a shift in species abundance in the FM group.

## Figures and Tables

**Table 1:** Cytokine plasma levels in FM patients with BMI ≤ 29.9 and ≥30 (Mann–Whitney U test).

Cytokine	BMI	Mean Cytokine Level (pg/mL)	*p*-Value
IFNγ	≤29.9	93.53	0.65
	≥30	102.49	
IL1 β	≤29.9	8.43	0.27
	≥30	18.94	
IL2	≤29.9	1.59	0.19
	≥30	3.49	
IL6	≤29.9	1.11	0.45
	≥30	1.75	
IL8	≤29.9	2.23	0.80
	≥30	1.53	
IL9	≤29.9	46.14	0.34
	≥30	33.02	
IL17A	≤29.9	5.52	0.27
	≥30	10.73	
IL18	≤29.9	77.74	0.76
	≥30	62.16	
TNFα	≤29.9	26.87	0.21
	≥30	42.29	

## Data Availability

The original data presented in the study are openly available in Rīga Stradiņš University Institutional Repository Dataverse at https://doi.org/10.48510/FK2/KAEYFM (accessed on 9 October 2024) and European Nucleotide Archive at https://www.ebi.ac.uk/ena/browser/view/PRJEB80379 (accessed on 9 October 2024).
